# HLA-VBSeq v2: improved HLA calling accuracy with full-length Japanese class-I panel

**DOI:** 10.1038/s41439-019-0061-y

**Published:** 2019-06-19

**Authors:** Yen-Yen Wang, Takahiro Mimori, Seik-Soon Khor, Olivier Gervais, Yosuke Kawai, Yuki Hitomi, Katsushi Tokunaga, Masao Nagasaki

**Affiliations:** 1 Graduate School of Information Sciences, Tohoku University, Sendai, Miyagi Japan; 2 Tohoku Medical Megabank Organization, Tohoku University, Sendai, Miyagi Japan; 3 The University of Tokyo, Bunkyo-ku, Tokyo Japan; 4 Toyama Genome Medical Science Project, National Center for Global Health and Medicine, Shinjuku-ku, Tokyo Japan; 5 Graduate School of Medicine, Tohoku University, Sendai, Miyagi Japan; 6 Center for the Promotion of Interdisciplinary Education and Research, Kyoto University, Sakyo-ku, Kyoto Japan; 7 Department of Microbiology, Hoshi University School of Pharmacy and Pharmaceutical Sciences, Shinagawa-ku, Tokyo Japan

**Keywords:** Next-generation sequencing, Data publication and archiving

## Abstract

HLA-VBSeq is an HLA calling tool developed to infer the most likely HLA types from high-throughput sequencing data. However, there is still room for improvement in specific genetic groups because of the diversity of HLA alleles in human populations. Here, we present HLA-VBSeq v2, a software application that makes use of a new Japanese HLA reference panel to enhance calling accuracy for Japanese HLA class-I genes. Our analysis showed significant improvements in calling accuracy in all HLA regions, with prediction accuracies achieving over 99.0, 97.8, and 99.8% in HLA-A, B and C, respectively.

## Introduction

The human leukocyte antigen (HLA) system is located within the 6p21.3 region on chromosome 6 and encodes the major histocompatibility complex (MHC) proteins that are essential to the immune system. HLA genes are associated with a wide range of disorders, including cancer, organ transplants, and autoimmune and infectious diseases^1–3^, and the current version of the IPD-IMGT/HLA Database, which indexes HLA sequences, contains more than 20,000 alleles of HLA subtypes, illustrating the extreme polymorphism of HLA alleles^4^. The accurate inference of HLA genotypes from whole-genome sequencing (WGS) data is therefore expected to significantly contribute not only to disease studies but also to fields such as pharmacogenomics. However, inferring HLA genotypes (HLA calling) remains challenging due to the substantial sequence similarity within the cluster and the exceptionally high variability of the loci.

In the past, we proposed a statistical method that infers the most probable HLA types from high-throughput sequencing data for each individual based on the optimal alignment of reads to the reference HLA sequences ^5^ and developed a computational tool called HLA-VBSeq^6^. While HLA-VBSeq uses the reference sequences of the IPD-IMGT/HLA Database for the alignment of read sequences, it is still possible to improve the prediction accuracy in specific populations since the allele distribution and haplotype frequencies of HLA genes largely depend on geographic location and genetic group^7,8^. In this study, we present an updated version, HLA-VBSeq v2, which adds a Japanese reference panel of class-I HLA genes (the ToMMo HLA panel^9^) to the IPD-IMGT/HLA Database to form a new reference panel.

To evaluate the performance of HLA-VBSeq v2 with the new reference panel, we compared the accuracy of HLA-VBSeq and HLA-VBSeq v2 by using WGS data in the Japanese population. Additionally, we compared the performance of HLA-VBSeq v2 with that of another HLA calling tool, HLA*PRG:LA, which calls for the alleles at G group resolution (a group of HLA alleles that have identical nucleotide sequences across the exons encoding the peptide-binding domains)^10^.

We also provide an exhaustive list of the ambiguous samples observed in our validation datasets. Ambiguous HLA alleles occurred because of the typing strategies. The PCR-SSOP-Luminex method only uses exons 2 and 3 for HLA class-I analysis (the HLA class-I region contains 8 exons), and this may result in more than one possible typing result within a given region. By using the WGS data, for which the HLA region is fully phased, HLA-VBSeq v2 displayed high performance in ambiguous samples; we therefore suggest that HLA-VBSeq v2 can be used to validate the typing results obtained with the PCR-SSOP-Luminex method.

The ToMMo HLA panel is a full-length Japanese reference panel of class-I HLA genes constructed from 208 samples at the Tohoku Medical Megabank Organization^9^. The ToMMo HLA panel consists of 139 alleles that were extended from knowns in the IPD-IMGT/HLA Database and includes 40 novel alleles compared with the closest subtypes in the IPD-IMGT/HLA Database. We added the ToMMo HLA panel to the IPD-IMGT3.31.0 reference sequences to create a new set of sequences to be used as a custom reference panel.

We used two independent datasets to evaluate and validate the prediction accuracy of HLA-VBSeq v2: the Tokyo Healthy Controls (THC) dataset and the Stevens-Johnson syndrome (SJS) dataset, using WGS data in both cases, i.e. with the HLA region fully phased. The THC dataset consists of 418 healthy Japanese individuals from the Tokyo area, and the SJS dataset consists of 117 Japanese individuals with cold medicine-related Stevens-Johnson syndrome with severe ocular complications.

To evaluate the performance of each calling tool, we calculated the prediction accuracy, defined as the percentage of inferences that are correct relative to the ‘true’ HLA types (the expected HLA types). In this study, we defined the ‘true’ HLA types by using the PCR-SSOP Luminex method and next-generation sequencing (NGS)-based HLA typing, which we implemented in both datasets. NGS-based HLA typing was performed with a NXType™ Class-I NGS HLA typing kit and the AllType™ NGS 11-loci Amplification Kit (Thermo Fisher Scientific, Waltham, MA, USA), which phased the full genes. The PCR-SSOP-Luminex method is a high-resolution genotyping method that combines polymerase chain reaction (PCR) and sequence-specific oligonucleotide probe (SSOP) protocols with the Luminex 100 xMAP technology at the HLA-A, B, and C loci, amplified by PCR on only exon 2 and exon 3^11^.

In this study, if PCR-SSOP-Luminex and NGS-based HLA typing gave the same result, it was considered to be the ‘true’ HLA types. By contrast, when samples displayed inconsistent typing results (‘ambiguous’ typing results), they were reexamined by Sanger sequencing-based typing (SBT). Since in all cases SBT gave the same result as NGS-based HLA typing, NGS-based HLA typing was considered to be the most reliable method to determine the ‘true’ HLA types.

In our analysis, we assessed the prediction accuracy of Japanese HLA calling from WGS data at the 2-field resolution, which specifies the amino acid sequence of the encoded protein. We evaluated two HLA alleles (either heterozygous or homozygous) in three HLA regions (HLA-A, B and C) or six HLA alleles in total for all individuals. Furthermore, we compared the performance of HLA-VBSeq v2 with that of HLA*PRG:LA, another HLA calling software application that infers alleles at G group resolution (the group of HLA alleles that have identical nucleotide sequences across the exon encoding the peptide-binding domains and are denoted by G at the 3-field resolution., e.g., A*01:01:01 G)^10^. HLA*PRG:LA reports a group of alleles for each gene at the 2-field resolution, but here we only used the first allele as the calling result.

The results obtained using HLA-VBSeq v2 show significant improvements in prediction accuracy in all HLA regions after including the ToMMo HLA panel in both the SJS and THC datasets (Table 1). Regarding the performance of HLA-VBSeq v2 compared to HLA*PRG:LA, though both programs achieved prediction accuracies of over 97.8%, HLA-VBSeq v2 displayed slightly better prediction accuracies for the HLA-A and HLA-C regions (99.0 and 99.8% vs. 98.1 and 98.4%, respectively) but lower performance for HLA-B genes (97.8 vs. 99.6%). HLA-VBSeq v2 displayed slightly better prediction accuracies for the HLA-A and -C regions in the THC and SJS datasets but lower performance for HLA-B genes. Moreover, in the SJS dataset, HLA-VBSeq v2 exhibited 100% prediction accuracy in the HLA-C region (Table 1).

**Table 1:** Comparison of the prediction accuracy between the software programs studied for each dataset and HLA gene region

Dataset	Gene (*n*)		HLA-VBSeq, %	HLA-VBSeq v2, %	HLA*PRG:LA, %
SJS	HLA-A	228	98.2	**99.1**	98.2
	HLA-B	228	92.5	97.8	**100**
	HLA-C	228	90.4	**100.0**	98.7
THC	HLA-A	836	98.4	**99.0**	98.1
	HLA-B	836	91.3	98.3	**99.6**
	HLA-C	818	92.1	**99.8**	98.4

*n* corresponds to the number of alleles in each dataset

*THC* Tokyo Healthy Controls data set, *SJS* Stevens–Johnson syndrome data set

Bold font corresponds to the highest prediction accuracy for each HLA gene

This study shows that including the ToMMo HLA panel substantially improves the calling accuracy of HLA class-I genes from WGS data in the Japanese population. In Table 2, we summarized the inference results obtained with HLA-VBSeq and HLA-VBSeq v2 that displayed inconsistencies with the ‘true’ genotype. We observed that the performance improved significantly with HLA-VBSeq v2 in some alleles, such as B*40:06, B*54:01, B*55:02 and C*14:03. For instance, HLA-VBSeq failed to correctly call C*14:03 (it was identified as C*14:02 in a high number of cases) in both datasets. The reference panel of HLA-VBSeq consists of reference sequences of C*14:02 and C*14:03 but not full genomic sequences (including exons, full introns and regulatory regions). By contrast, HLA-VBSeq v2 uses the ToMMo HLA panel, which contains the full-length reference sequences of C*14:02 and C*14:03 for the Japanese population, and therefore identified C*14:03 correctly. This indicates that full genomic sequences are highly informative in HLA calling and that using HLA-VBSeq v2 leads to more accurate calling results in the Japanese population.

**Table 2:** Details of the calling results inconsistent with the “true” HLA types. “fail” indicates that the program failed to reach an appropriate calling result

		Discordance (*n*)					
Dataset	gene	True type (*n*)		HLA-VBSeq		HLA-VBSeq v2	
SJS	A	A*02:01	33	A*02:06	2	A*02:06	1
		A*24:02	55	fail	1		
		A*26:02	3			A*26:01	1
		A*31:01	18	A*33:03	1		
	B	B*15:01	8	B*46:01	1		
		B*15:27	1			B*15:01	1
		B*40:06	8	B*40:02	5		
		B*51:01	21	B*51:02	4	B*51:02	3
		B*54:01	8	B*55:01	1		
		B*55:02	4	B*55:01	2		
		B*56:01	3	B*55:01	3		
		B*59:04	1	B*59:01	1	B*59:01	1
	C	C*01:02	40	C*14:02	1		
		C*04:82	1	C*04:01	1		
		C*14:03	28	C*14:02	20		
THC	A	A*02:15 N	1			A*02:07	1
		A*03:02	1			A*03:01	1
		A*11:02				A*11:01	1
		A*24:02	314	A*26:01	1		
		A*24:08	1			A*24:02	1
		A*24:20	10			A*24:02	1
		A*26:01	67	A*02:06	1	A*02:06	1
				A*25:01	1		
				A*33:03	4		
		A*26:02	12	A*24:02	2	A*24:02	1
		A*26:03	22	A*24:02	1		
				A*26:01	1		
				A*66:01	1		
		A*26:05	1	A*26:01	1	A*26:01	1
	B	B*07:169	1	B*07:02	1	B*07:02	1
		B*15:01	71	B*46:01	1		
		B*15:07	5	B*15:01	1		
		B*15:11	5	B*07:02	1		
				B*15:01	1		
				B*40:02	1		
				B*46:01	1		
		B*15:27	1			B*15:01	1
				B*46:01	1		
		B*15:28	1	B*15:428	1	B*15:428	1
		B*35:01	67	B*39:01	1	B*39:01	1
		B*40:02	57	B*40:356	1		
		B*40:06	34	B*40:01	1		
				B*40:02	20		
				B*40:04	2		
				B*44:03	1		
				B*46:01	1		
		B*40:52	1			B*40:01	1
				B*40:02	1		
		B*51:01	70	B*15:01	1	B*15:01	1
						B*40:01	1
				B*51:02	1	B*51:02	4
						B*54:01	1
		B*52:01	80	B*07:02	1		
				B*51:01	1		
						B*51:02	1
				B*52:54	1		
		B*54:01	64	B*15:18	1		
				B*35:01	2		
				B*40:02	2		
				B*44:02	1		
				B*44:03	3		
				B*46:01	1		
				B*55:01	7		
		B*55:02	20	B*35:01	1		
				B*40:01	1		
				B*51:01	2		
						B*54:01	1
				B*55:01	3		
				B*67:01	1		
		B*56:01	5	B*55:01	4		
		B*67:01	11	B*39:01	2		
	C	C*01:02	135	C*08:01	1		
		C*03:02	2	C*03:04	2		
		C*03:03	111	C*03:04	1	C*03:04	1
				C*07:02	1	C*07:02	1
		C*04:82	10	C*04:01	8		
		C*08:22	2	C*08:01	1		
		C*14:03	67	C*07:02	1		
				C*12:02	1		
				C*14:02	49		

Bold font highlights an example (mentioned in the text) for which including the ToMMo HLA panel allowed identification of the correct HLA type

Ambiguous allele combinations occurred in all HLA loci; a possible reason for this may lie in the presence of heterozygous sequences, i.e., the presence of more than one possible pair of alleles within the region analyzed or the existence of a polymorphism outside of the region analyzed^12^. Because the PCR-SSOP-Luminex method only covers exons 2 and 3 for HLA class-I analysis (there are 8 exons in HLA-A, B and C regions)^11^, we also observed some ambiguous samples in our datasets (Table 3).

**Table 3:** Typing results between the Luminex method and NGS-based HLA typing for all inconsistent samples. Bold font corresponds to the typing results from NGS-based HLA typing

	HLA typing			HLA calling		
Dataset	Sample	NGS-based^a^	Luminex	HLA-VBSeq	HLA-VBSeq v2	HLA*PRG:LA
SJS	SJS01	B*59:04	B*59:01	B*59:01	B*59:01	B*59:04
	SJS02	C*07:06	C*07:01	C*07:06	C*07:06	C*07:01
	SJS03	C*08:22	C*08:01	C*08:22	C*08:22	C*08:01
	SJS04	C*04:82	C*04:01	C*04:01	C*04:82	C*04:01
THC	THC01	A*02:15 **N**	A*02:07	A*02:15 **N**	A*02:07	A*02:07
	THC02	A*11:02	A*11:01	A*11:02	A*11:01	A*11:02
	THC03	B*07:169	B*07:02	B*07:02	B*07:02	B*07:02
	THC04	C*08:22	C*08:01	C*08:22	C*08:22	C*08:01
	THC05	C*08:22	C*08:01	C*08:01	C*08:22	C*08:01
	THC06	C*04:82	C*04:01	C*04:01	C*04:82	C*04:01
	THC07	C*04:82	C*04:01	C*04:82	C*04:82	C*04:01
	THC08	C*04:82	C*04:01	C*04:01	C*04:82	C*04:01
	THC09	C*04:82	C*04:01	C*04:01	C*04:82	C*04:01
	THC10	C*04:82	C*04:01	C*04:01	C*04:82	C*04:01
	THC11	C*04:82	C*04:01	C*04:82	C*04:82	C*04:01
	THC12	C*04:82	C*04:01	C*04:01	C*04:82	C*04:01
	THC13	C*04:82	C*04:01	C*04:01	C*04:82	C*04:01
	THC14	C*04:82	C*04:01	C*04:01	C*04:82	C*04:01
	THC15	C*04:82	C*04:01	C*04:01	C*04:82	C*04:01

^a^These typing results were used as the “true” types

Table 3 shows that HLA-VBSeq v2 provided outstanding performance in ambiguities in the Japanese population. For instance, since C*04:01 and C*04:82 have identical nucleotide sequences in exons 2 and 3, it is impossible to distinguish them only by analyzing exon-2 and -3 sequences, leading to an ambiguous result. Because the ToMMo HLA panel includes the full-length reference sequences of C*04:82, HLA-VBSeq v2 distinguished C*04:01 from C*04:82 and made correct calls.

Table 3 also shows that in most instances, HLA*PRG:LA and Luminex inferred the same allele type when dealing with ambiguous cases. Similarly, NGS-based HLA calling and HLA-VBSeq v2 reached the same result in a high number of cases. The reason why HLA*PRG:LA and Luminex tend to provide the same calling results likely lies in the design of HLA*PRG:LA to call for the alleles at G group resolution^10^, which means that the calling methods focus on exons 2 and 3. By contrast, HLA-VBSeq v2 is designed for 8-digit resolution and includes the full-length HLA sequences for the analysis, making it more likely that the results will coincide with those from NGS-based HLA typing (“true” HLA types). Because of these differences, the performance of HLA calling in ambiguous cases varies greatly depending on the software application used. Given the high performance of HLA-VBSeq v2 in ambiguities, we suggest reexamining PCR-SSOP-Luminex typing results with HLA-VBSeq v2 to obtain more reliable results regarding the correct HLA type in the Japanese population.

In conclusion, the addition of a reference panel for the Japanese population was highly effective for improving the calling accuracy of HLA class-I genes from WGS data in the Japanese population. This solution is promising for the inference of HLA class-II genes and other highly diverse regions, e.g., killer-cell immunoglobulin-like receptors (KIRs). Additionally, this study suggests that the ToMMo HLA panel is a valuable resource whose use is not limited to HLA-VBSeq v2 but could be expanded to other HLA calling tools.

## Data Availability

HLA-VBSeq v2 is available from the following URL: http://nagasakilab.csml.org/hla/.

## References

[1] Shukla SA (2015). Comprehensive analysis of cancer-associated somatic mutations in class I HLA genes. Nat Biotechnol.

[2] Flomenberg N (2004). Impact of HLA class I and class II high-resolution matching on outcomes of unrelated donor bone marrow transplantation: HLA-C mismatching is associated with a strong adverse effect on transplantation outcome. Blood.

[3] Shiina T, Hosomichi K, Inoko H, Kulski JK (2009). The HLA genomic loci map: expression, interaction, diversity and disease. J Hum Genet.

[4] Robinson J (2015). TheIPD and IPD-IMGT/HLADatabase: allele variant databases. Nucleic Acids Res.

[5] Nariai N, Hirose O, Kojima K, Nagasaki M (2013). TIGAR: transcript isoform aboundance estimation method with gapped alignment of RNA-Seq data by variational Bayesian inference. Bioinformatics.

[6] Nariai N (2015). HLA-VBSeq: accurate HLA typing at full resolution from whole-genome sequencing data. BMC Genomics.

[7] Gourraud PA (2014). HLA diversity in the 1000 genomes dataset. PloS One.

[8] Pappas DJ, Tomich A, Garnier F, Marry E, Gourraud PA (2015). Comparison of high-resolution human leukocyte antigen haplotype frequencies in different ethnic groups: Consequences of sampling fluctuation and haplotype frequency distribution tail truncation. Hum Immunol.

[9] Mimori Takahiro, Yasuda Jun, Kuroki Yoko, Shibata Tomoko F., Katsuoka Fumiki, Saito Sakae, Nariai Naoki, Ono Akira, Nakai-Inagaki Naomi, Misawa Kazuharu, Tateno Keiko, Kawai Yosuke, Fuse Nobuo, Hozawa Atsushi, Kuriyama Shinichi, Sugawara Junichi, Minegishi Naoko, Suzuki Kichiya, Kinoshita Kengo, Nagasaki Masao, Yamamoto Masayuki (2018). Construction of full-length Japanese reference panel of class I HLA genes with single-molecule, real-time sequencing. The Pharmacogenomics Journal.

[10] Dilthey AT (2016). High-accuracy HLA type inference from whole-genome sequencing data using population reference graphs. PLoS Comput Biol.

[11] Itoh Y (2005). High-throughput DNA typing of *HLA-A*, *-B*, *-C*, and *-DRB1* loci by a PCR-SSOP-Luminex method in the Japanese population. Immunogenetics.

[12] Adams SD (2004). Ambiguous allele combinations in HLA Class I and Class II sequence-based typing: when precise nucleotide sequencing leads to imprecise allele identification. J Transl Med.

